# Detecting simulated skew deviation using a smartphone eye-tracking application (EyePhone): a feasibility study

**DOI:** 10.3389/fneur.2026.1860824

**Published:** 2026-07-21

**Authors:** Rajvi Babaria, Pouya Barahim Bastani, Hector Rieiro, Shervin Badihian, Jorge Otero-Millan, Ali Saber Tehrani

**Affiliations:** 1 Johns Hopkins University School of Medicine, Baltimore, MD, United States; 2 Department of Neurology, University of Michigan, Ann Arbor, MI, United States; 3 Neurological Institute, Cleveland Clinic, Cleveland, OH, United States; 4 Herbert Wertheim School of Optometry and Vision Science, University of California, Berkeley, Berkeley, CA, United States

**Keywords:** acute vestibular syndrome (AVS), eye (saccade), eye movements, posterior circulation stroke, smartphone eye-tracking application

## Abstract

**Introduction:**

Strokes presenting with acute dizziness (i.e., vestibular strokes) are four times more likely to be misdiagnosed compared with strokes presenting with typical symptoms such as weakness. The HINTS eye exam (Head Impulse test, Nystagmus, and Test of Skew) can improve the diagnostic accuracy for vestibular strokes with 98% Sensitivity and 97% specificity. As part of the HINTS battery, the test of skew evaluates the vertical misalignment of the eyes of a vestibular cause. Skew deviations of >5 diopters are highly specific for stroke. However, as experts who can accurately detect skew deviation are not readily available in the ED, there is a need for accessible methods to quantify skew deviation. We sought to investigate the feasibility of detecting and quantifying simulated skew deviations using our smartphone eye-tracking application (EyePhone).

**Methods:**

We enrolled healthy volunteers and recorded their eye movements using EyePhone. To simulate skew deviation, we used two targets on a wall 1 m away. As the examiner performed the cross-cover test, participants shifted their gaze between the upper and lower target whenever the occluder moved between their eyes, mimicking the refixation saccades seen in skew deviation. We began with targets separated by 20 cm at 1 m (representing 20 prism diopters of skew) and gradually reduced this distance until the targets overlapped, indicating the absence of skew. We used videos recorded by EyePhone using the front camera of an iPhone placed within 40 cm of the eyes. We developed in-house code using features extracted with Google’s Mediapipe to analyze the eye movements after automatically detected eye uncoverings.

**Results:**

The correlation between the EyePhone measured and the designed misalignment suggests that EyePhone may be capable of detecting skew deviations of ≥5 diopters in a controlled experimental setting. Analysis showed a Spearman correlation of 0.93 (CI: 0.90–0.95) between the EyePhone measurements and the design-induced skew deviations. The calculated area under the receiver operator curve for discriminating skews≥5 diopters from smaller ones was 0.90.

**Discussion/conclusion:**

These findings suggest that EyePhone may potentially serve as a practical tool for the detection of clinically significant skew deviation following further validation in clinical groups.

## 1 Introduction

Of the ~5 million annual visits to emergency departments for dizziness and vertigo, approximately 3–5% are due to cerebrovascular accidents (1, 2). The rapid and accurate diagnosis of stroke is imperative for patients to receive acute treatments (3). For instance, cerebellar strokes that are missed during an initial ED visit can lead to an eightfold increased risk of death (4). One type of stroke is posterior circulation stroke (PCS), which results from poor perfusion of brainstem, cerebellum, thalamus, and/or occipitoparietal lobe (5). The identification of PCS patients presents a serious clinical obstacle for emergency physicians as symptoms are typically isolated, and focal neurologic signs are typically not present (4). Of the ~130,00 to 220,000 patients with stroke presenting with dizziness or vertigo to the emergency department, it is estimated that ~45,000 to 75,000 are misdiagnosed (6).

Acute vestibular syndrome (AVS) is acute continuous dizziness within 24 h of symptoms onset and can be due to a peripheral or a central cause (6). Features of the syndrome are suggestive of new, ongoing dysfunctions in the vestibular system, including nystagmus and vomiting (6). Peripheral causes include inner ear or vestibular nerve abnormalities, while central causes involve lesions in the brainstem or cerebellum. One predictor of a central cause of AVS is skew deviation, which is a vertical ocular misalignment of a vestibular cause, and often manifests as diplopia (7, 8). Skew is typically detected via cover-uncover test or cross-cover test (7). Skew deviation is associated with not only vertical eye deviation, but may also be accompanied with ocular counter-roll and head tilt (8). Skew deviation can be subtle and, therefore, detection of skew in the emergency setting is challenging.

The HINTS (head impulse, nystagmus, test of skew) eye exam can differentiate between a peripheral or a central cause in patients with AVS (9). The assessment involves examining eye movements for spontaneous or gaze-evoked nystagmus, performing a head impulse in which the head is rapidly turned to interrogate the ipsilateral vestibulo-ocular reflex, and assessing misalignment of the eyes (9). However, abnormalities of eye movements are subtle and challenging to recognize by the naked eye without specialized training.

Video oculography goggles, such as the FDA-approved ICS goggles (Natus Medical, WI), can quantify eye movements in clinical settings, but these devices are expensive, require neurovestibular experts for interpretation, and are not commonly available in the emergency department. In contrast, smartphones pave the way for efficient eye movement analysis, as they are ubiquitous and capable of recording under varying light conditions (10). Due to their increasing availability in the consumer market, smartphones increase the accessibility to eye movement recording technology.

In a previous study, our group developed a smartphone application, EyePhone, that records and quantifies eye movements without additional hardware. That study assessed the application’s ability to detect nystagmus in varying directions and compare its accuracy with the video oculography goggles (11). To further investigate the feasibility and possible utility of the application, this study focuses on assessing the ability of the application to detect simulated skew deviation, an important marker of stroke.

## 2 Methods

We enrolled healthy volunteers (*n* = 10; 90% male, average age 30.2 ± 6.0) and recorded eye movements using the EyePhone application on an iPhone 13 ProMax (Apple) with a triple 12MP camera system. The application is compatible with any phone with face ID, and we used the most up-to-date version of the iOS at the time of the recording (iOS 14.8). The application is maintained by our study team, and it is currently compatible with the latest iOS version. The recordings were obtained between January and September 2022 from volunteers with no known history of eye movement or vestibular disorders.

The volunteers included in this study were 18 years or older and had no history of vestibular or ocular motor disorders. The exclusion criteria included participants having abnormal eye movements before recording and any visual disturbances that render participants unable to follow the calibration targets.

All recordings were performed in a well-lit room, and the same targets and devices were used to obtain comparable recordings. The frame rate/sampling frequency was 1,080 and 60 FPS. The recordings involved an initial calibration phase followed by recording participants’ eye movements as they focused on an optokinetic stimulus.

Informed written consent was obtained from all participants. The Institutional Review Board at Johns Hopkins approved the study protocol (#IRB00258938).

### 2.1 Calibration

Eight horizontal targets were placed on the wall at ± 5 degrees (8.75 cm), ± 10 degrees (17.5 cm), ± 15 degrees (26.25 cm), and ± 25 degrees (43.75 cm) from the center. Positive values represent the right and negative values for the left with respect to the visual field at 1 m distance. Six vertical targets were placed at ± 5 degrees (8.75 cm), ± 10 degrees (17.5 cm), and ± 20 degrees (35.0 cm) from the center in the positive/upwards and negative/downwards directions. The center of a chair was placed 1 m away from a central target placed at eye level on the wall.

**Figure 1:**
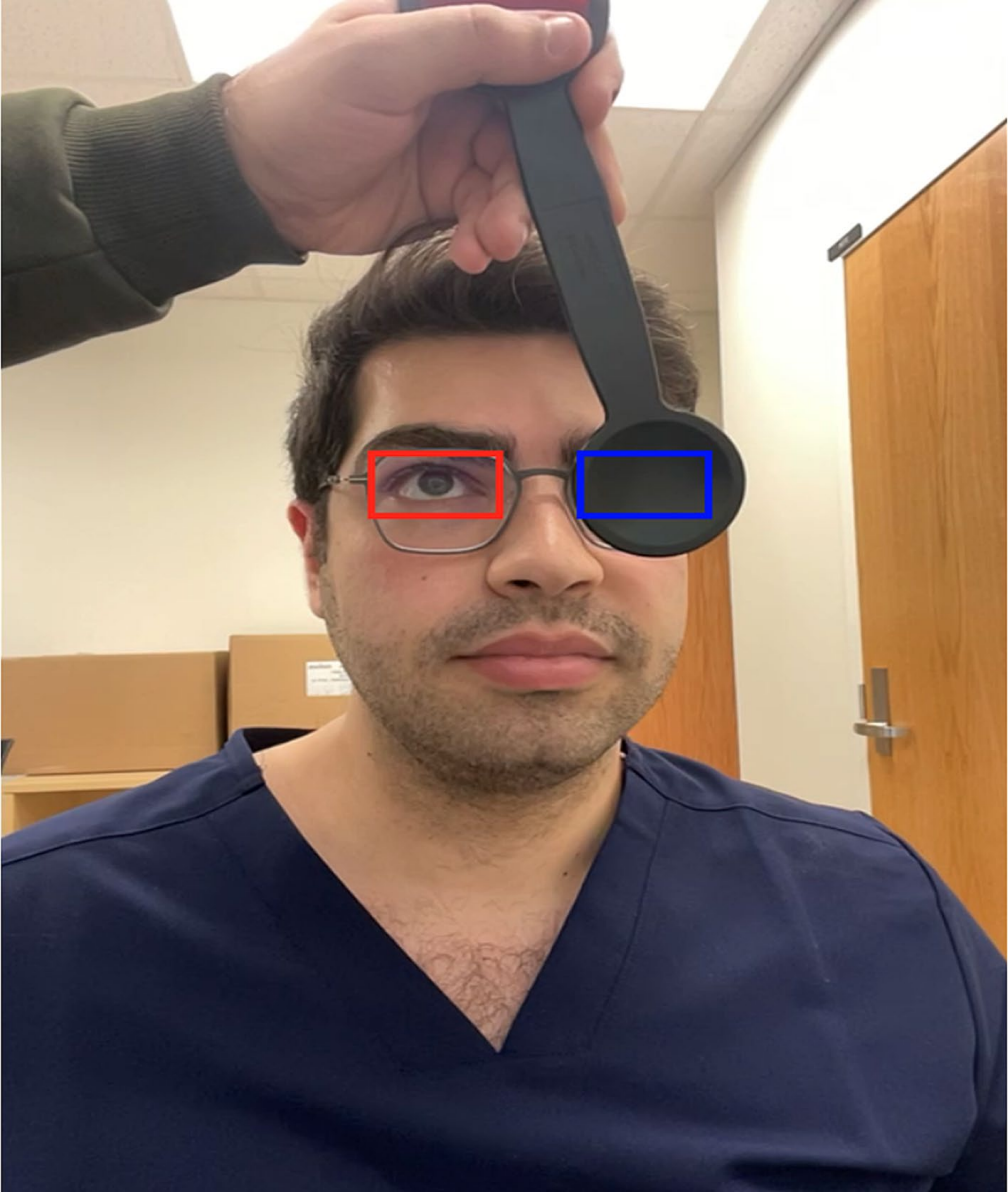
Occluder covering one eye for cross-cover test.

The participants were seated on a chair and the center of the chair was 1 m away from the wall. The chair’s height was adjusted to ensure the central target would be at the center of the participant’s visual field. We did not restrain the participants’ heads, but we advised them to limit any head movement to obtain accurate calibration recordings. The participant’s distance of 1 m from the target was confirmed by laser. The smart phone was placed in a tripod 40 cm away from the participant’s face, and it was kept at a slight angle to not obstruct the vertical tape markers (fixation targets) in the negative/downwards direction (see Figure 1). We obtained 2 separate calibration recordings, 1 for the horizontal and 1 for the vertical calibration. If the participants were not able to perform the calibration as they were instructed, the operator discarded the recording and reinstructed the participant to obtain a new recording.

### 2.2 Skew test

To simulate a skew deviation, two targets (tape markers) were placed vertically on a wall, 20 cm apart, with one at eye level. The participants sat exactly 1 m away from the targets (we used a laser set-up to ensure proper alignment for each individual). They were asked to fixate on the lower target. A black occluder was used to perform the cross-cover test. The occluder was held above the participant’s head for 3 s before being moved to the other eye. After the occluder was moved from one eye to the other, the examiner waited for 1 s, and the participant was then asked to fixate on the target 20 cm above. To maximize consistency throughout the testing process, we used an interval timer with two distinct alarms, one for crossing the occluder and the other for switching the fixation. This created an induced vertical misalignment of 20 prism diopters. This was repeated 20 times, where the participant looked at an alternative target each time, resulting in 10 uncoverings for each eye. Each trial and the amplitude of the recorded steps were documented with the application. Lastly, the distance between targets was reduced to 15, 10, 5, 3, 2, and 0 cm, and the process was repeated.

The correspondence between the physical position of the targets was estimated and validated both clinically (prism bars by a neuro-ophthalmologist) and by ICS-impulse video goggles (Natus, WI), and exact equal skew results were found in every single assessment. These calculations were performed before any EyePhone data was collected.

### 2.3 Video processing and eye movement analysis

We used Google’s Mediapipe suite to extract facial landmarks frame by frame (12). We stored information on eye luminance, iris position and nose coordinates. As a first step, we subtracted the nose position form the detected iris position for both eyes to account for head translation during the recording We used the luminance around the eyes region to detect the covering and uncovering of the eyes: since the occluder was black, the luminance of these regions was lower when the eyes were covered. To eliminate false transitions between covered/uncovered states, we removed state transitions with durations lower than 1 s. The resulting uncover times were individually validated against the video by the authors.

We calibrated our eye measurements following a protocol similar to the one described in our previous EyePhone publication (13). In that study, we found that, under similar recording conditions, an averaged calibration routine across subjects gives enough precision for measurements in the range we are testing here, so we used the same coefficients from that study from all recordings of all subjects, with no changes between subjects or repetitions.

**Figure 2:**
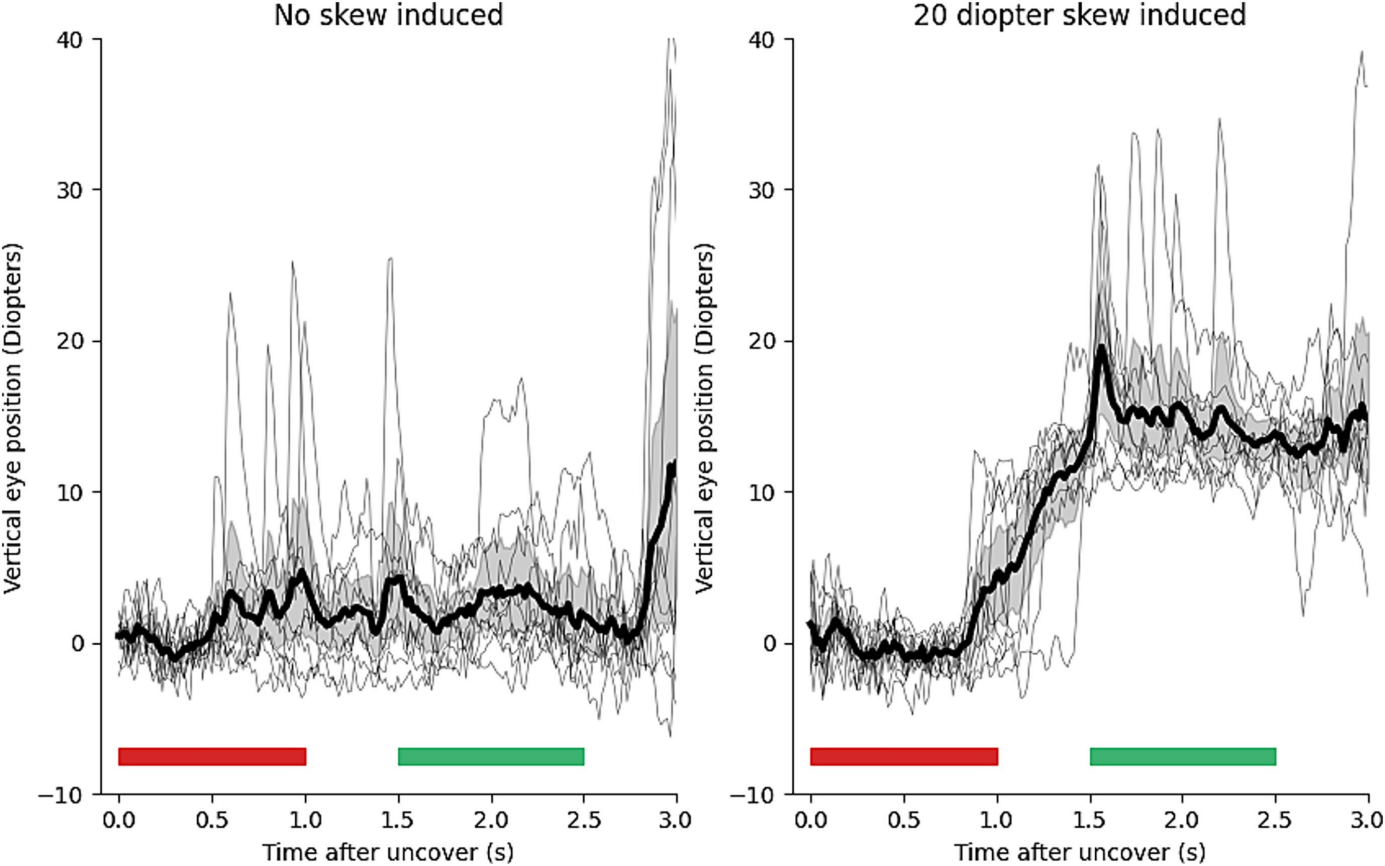
Raw representative eye-tracking correlograms for one subject and two induced displacements. Thin lines show the individual repetitions, thick lines show the average, and red and green lines indicate the periods averaged to calculate displacement.

After detecting the exact times the eyes were uncovered, we captured and averaged the pre- and post-saccadic eye positions. We calculated the saccade displacement by subtracting the average vertical position in the second before the saccade from the average vertical position 500 ms after the saccade, to account for reaction time and saccade duration (Figure 2). All analyses were performed using custom Python 3 code.

### 2.4 Statistical analysis

We initially performed a correlation analysis to determine general agreement between the theoretical induced skew values. We then performed a mixed-effects linear model regression to obtain precise estimates of the effect of induced skew in our measurements, as well as intersubject variability. In this model, we used the induced skew as fixed effect, and the individual subjects as random effect. We fitted two models, one with a single slope and another with a different slope for each subject, and selected the latter based on the difference between their Akaike Information Criterion (*ΔAIC = 130.32*).

Since we performed 10 consecutive uncoverings for each induced skew value per subject, we were interested to know how reliable each individual measurement was, as well as how many measures we needed to average in order to get reliable results. We estimated this by performing an Intraclass Correlation analysis (ICC), following previously described guidelines (14). In short, we calculated ICC(1, 1), ICC(A, 1), and ICC(C, 1) over the whole dataset to estimate the reliability of a single eye uncovering. Then, we calculated ICC(1, k), ICC(A, k), and ICC(C, k) by averaging the first *k* repetitions of each stimulus and did this for 2 ≤ k ≤ 10 (14). We used an ICC threshold of 0.75 or higher as criterion for good reliability.

We assessed our methodology’s predictive potential using Receiver Operating Characteristic (ROC) analysis. We performed two such analyses: in the first one we compared induced vertical displacement for each tested size against the zero displacement condition to obtain the discriminability of different skew sizes. In the second one, we classified our displacements between clinically relevant (those over five diopters), and clinically irrelevant, and tested the system’s capability to discriminate those.

## 3 Results

### 3.1 Cross-cover test recording setup and correlograms

**Figure 3:**
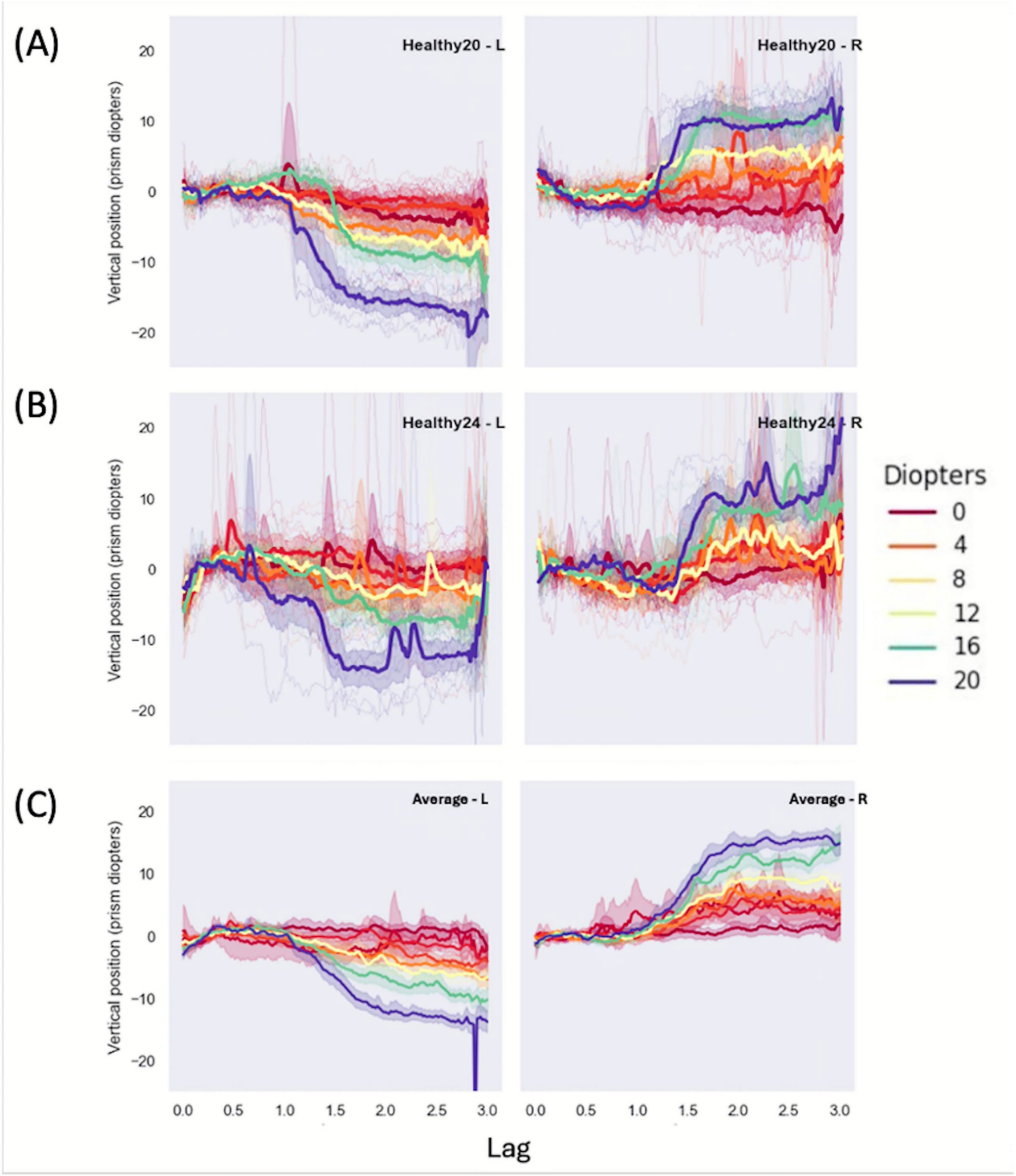
**(A,B)** Combined correlograms showing substantially larger and more sustained deviations at higher induced displacements (>5 prism diopters) and minimal vertical displacement at lower prism diopter values in both the left and right eye after cross-cover test for two healthy subjects **(C)** Average correlogram (*n* = 10) demonstrating consistent detection of induced skew deviation.

Figure 3 shows cross-cover test eye position correlograms from two subjects followed by an average correlogram of all subjects. There are small displacements present for smaller diopters (<5), with larger changes occurring for displacements greater than 5 diopters, emphasizing a more pronounced deviation.

### 3.2 Agreement analysis and diagnostic performance

**Figure 4:**
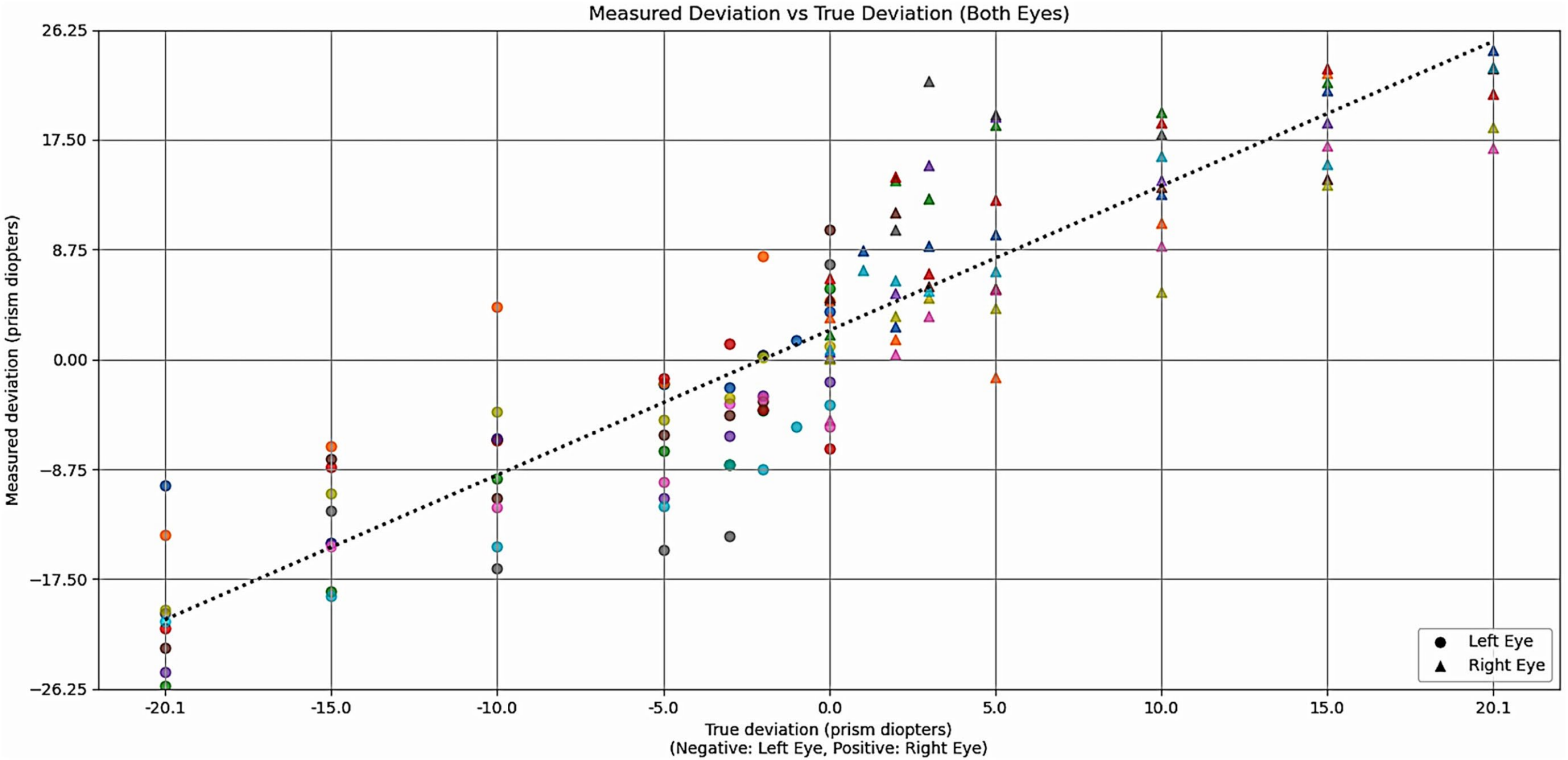
Strong correlation between the measured deviation from the EyePhone and the true (designed) deviation for the left and right eye; spearman correlation of 0.93 (CI: 0.90–0.95).

Figure 4 shows a positive correlation between the measured and true deviation. However, the predominant scattering of the data points around the fitted line suggests agreement between the measurements from EyePhone and the induced deviations under a controlled experimental setting. The Spearman correlation between the EyePhone measurements and the design induced skew deviations is 0.93 (CI: 0.90–0.95, *p* < 0.01).

**Figure 5:**
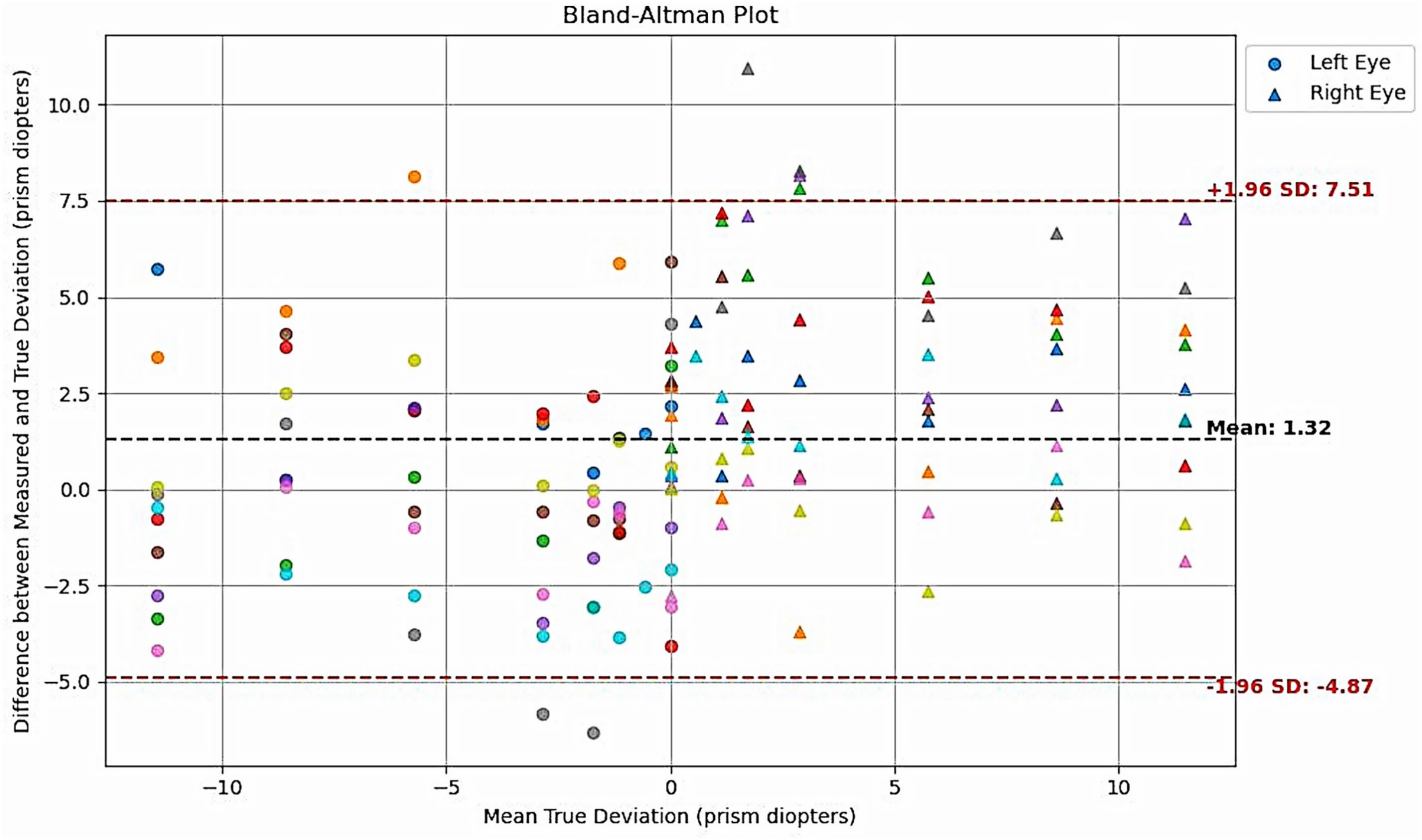
Bland–Altman plot showing the consistency of the EyePhone application’s estimations.

The Bland–Altman plot in Figure 5 shows the agreement between the EyePhone’s measured deviation and the true deviation. The mean difference is indicated by the dotted black line at 1.32 prism diopters, highlighting the application’s slight tendency to overestimate the true deviation. The spread of the data points suggests that most of the participants’ data falls within the limits of agreements, which are indicated by the red dashed lines. This suggests the application’s ability to perform within that measurement range.

**Figure 6:**
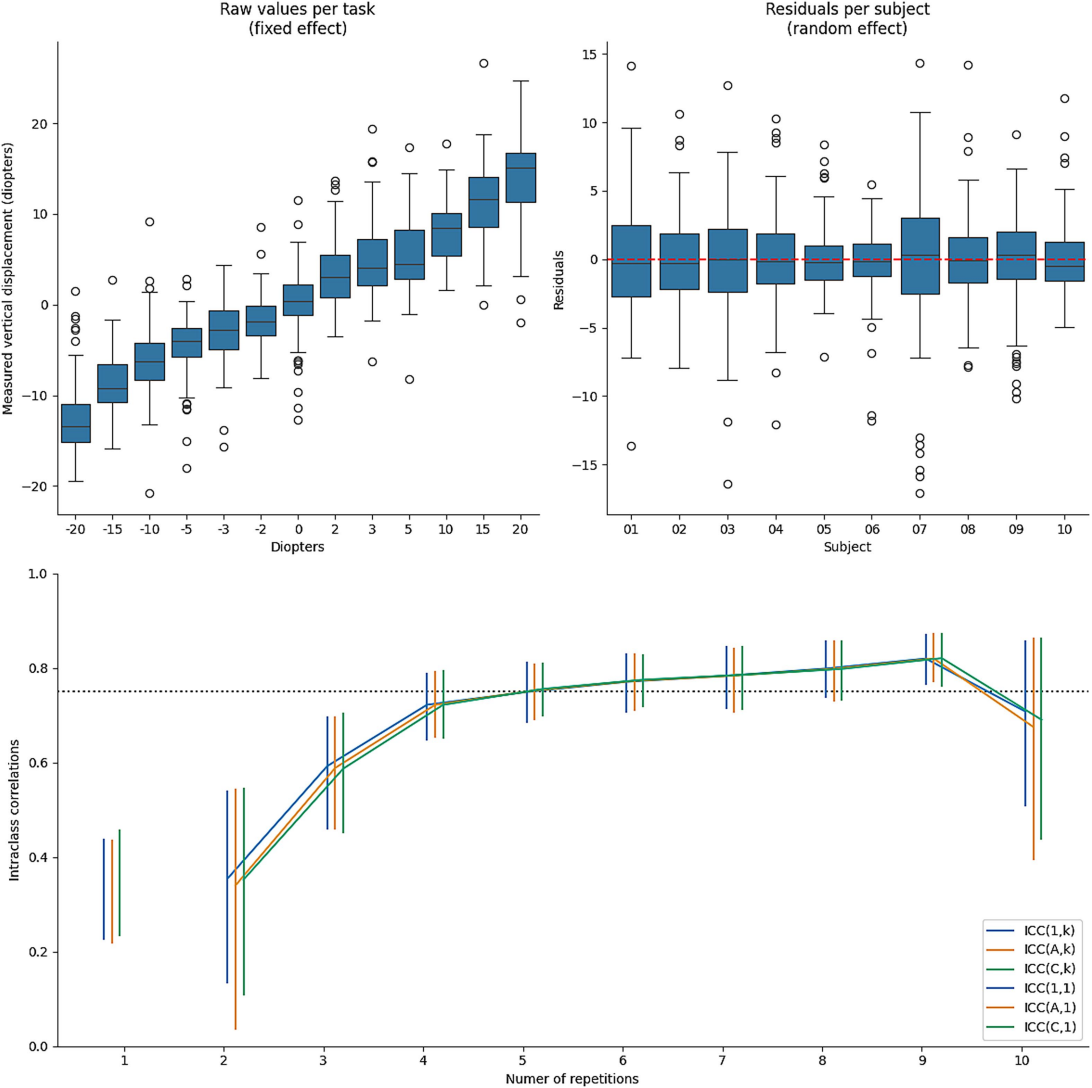
Linear mixed effects model measuring the effect of the induced skew on the measured eye vertical displacement. Top row, left panel: Measure of the main effect. Right panel: model residuals for each individual subject (random effect). Bottom row: intraclass correlation analysis. The different colored traces represent the value of the different ICC(1/A/C, *) coefficients. The points at *n = 1* show the reliability of individual measurements per subject (*ICC*(*1/A/C,1*)). The points for *n > 1* indicate the reliability of averaging *n* repeated measures. The horizontal dotted line marks the 0.75 threshold generally considered an indicator of good reliability.

Figure 6 and Table 1 show the results of our mixed-effects linear regression model, as well as the ICC analysis. We found a strong effect of induced diopter displacement on our measurements (*p* < 0.001) (Figure 6, top left). The residual plot (Figure 6, top right) shows residuals for all subjects centered around zero, and similar variability across the sample, indicating good model fitting.

**Table 1:** Summary table of results for the mixed effects linear model.

Parameter	Coefficient	Standard error	*z*	*P* > |*z*|	95% CI
Intercept	0.857	0.326	2.63	0.009	(0.218, 1.496)
Diopters	0.694	0.041	16.741	<0.001	(0.613, 0.7775)
Subject variance	0.968	0.0144			
Subject × diopters covariance	0.066	0.014			
Diopters variance	0.016	0.002			

The ICC analysis (Figure 6, bottom panel) shows the results of the ICC analysis. The first thing to notice is that ICC (1), ICC(A), and ICC(C) are nearly identical, pointing to no significant biases between measurements (14). For single repetitions, the ICCs are all below 0.4, indicating a single measurement is not reliable enough, and several observations need to be averaged. We see in the plot that around 5 measurements (i.e., eye uncoverings) are required in order to get a reliable estimate.

**Figure 7:**
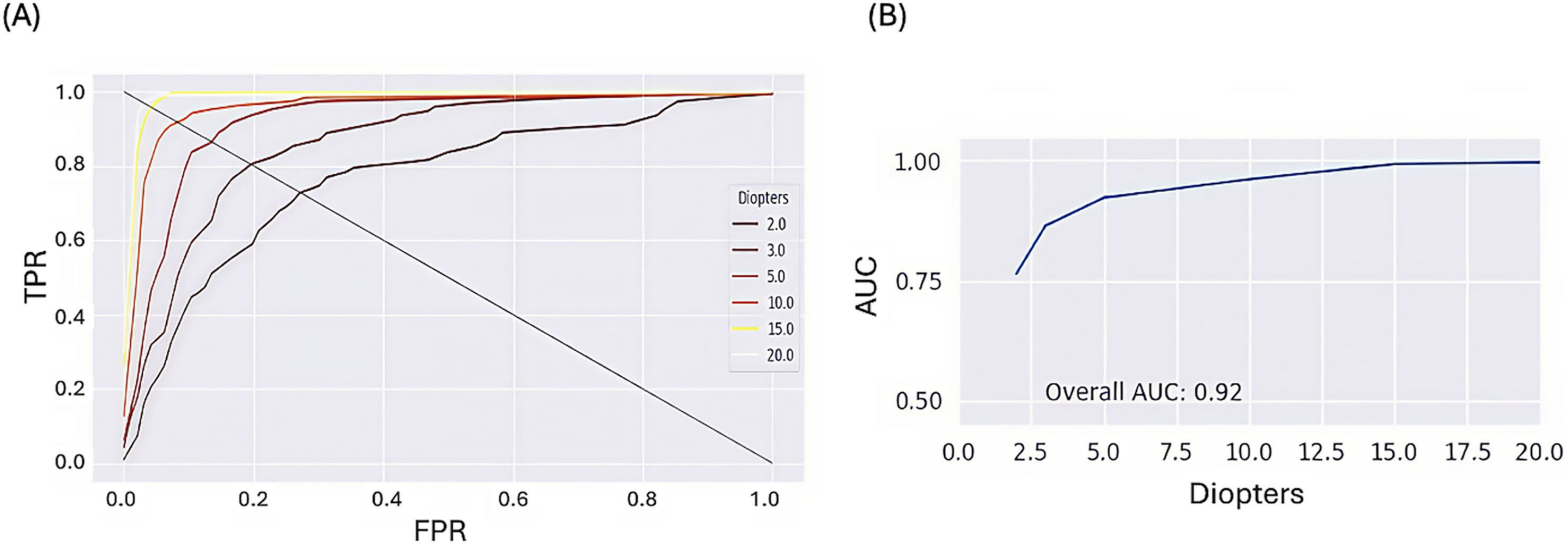
ROC analysis for detection of induced skew versus no induced skew based on trial setup; **(A)** *x*-axis represents the FPR (proportion of no skew detection trials classified as skew) and *y*-axis represents the TPR (proportion of skew detection trials correctly classified); **(B)** improved detection performance as prism diopters increases; ROC, receiver operating characteristic curve; AUC, area under the curve; FPR, false positive rate; TPR, true positive rate.

Figure 7 shows the receiver-operating characteristic (ROC) for discriminating skews of varying magnitudes. The ROC evaluates the model’s performance across the varying diopter values, plotting the false positive rate (FPR) and the true positive rate (TPR). The quick rise of each curve suggests preliminary discriminatory capability due to a high TPR and a low FPR. As expected, the higher diopter values (e.g., 15 and 20) exhibit better performance than the lower diopters.

**Figure 8:**
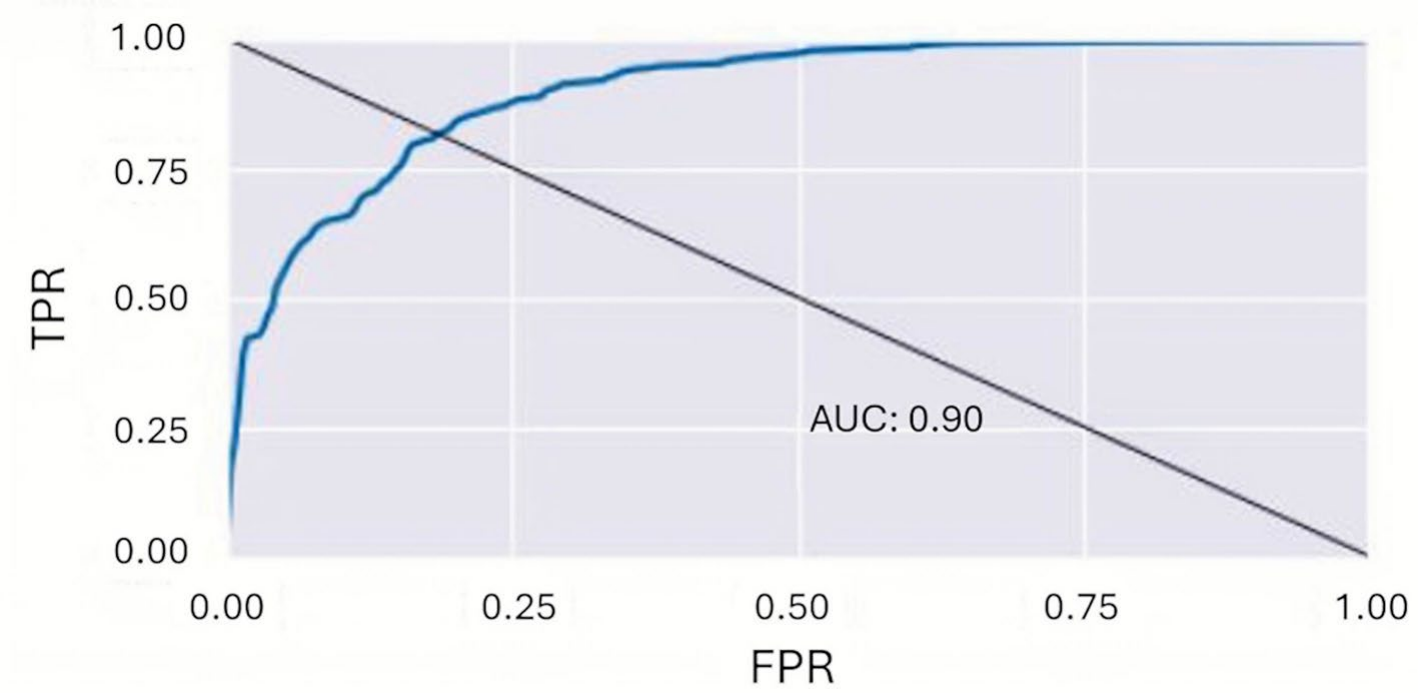
Strong AUC of 0.9 for discriminating 5 diopters based on the clinical threshold for vertical misalignment.

Figure 8 shows the ROC curve for detecting skews above 5 diopters. The steep curve suggests the potential ability of EyePhone to achieve a high true positive rate while maintaining the low false positive rate, which is evidence of its ability to detect simulated vertical misalignments in this controlled setting.

## 4 Discussion

This study represents an exploratory step in the overarching goal of our team in using a handheld device to potentially capture and quantify various components of the HINTS exam. We previously indicated the accuracy of our application in quantifying the head impulse test (11) and induced nystagmus (13). Our previous study indicated that the EyePhone application can accurately identify optokinetic nystagmus (OKN) at different velocities and directions and quantify the velocity of the OKN (15). This study explores the potential ability of EyePhone to detect and quantify simulated skew deviation. Along with optokinetic nystagmus, skew deviation is another critical marker for stroke. The application demonstrated promising preliminary feasibility results for the detection of vertical misalignment in a controlled research setting. This suggests that the application may potentially have future clinical utility in the diagnosis of strokes, specifically in emergency department settings, upon further validation studies.

Although skew deviation can be evaluated using a bedside cover-uncover or cross-cover test, the accurate detection and interpretation of subtle eye movements is challenging in emergency department settings. This challenge is specifically relevant in patients with acute dizziness or vertigo, and within this group, cerebrovascular causes are often missed during the initial assessment (2). Diagnostic errors are prevalent among patients presenting with acute dizziness or vertigo and an estimated 45,000–75,000 cases of vestibular stroke are initially misdiagnosed in United States emergency department each year (6). As posterior circulation strokes often lack evident focal neurological deficits, there is a need for more accurate bedside methods to assess eye movements.

The HINTS examination has shown to be a valuable bedside tool for differentiating between peripheral and central causes of AVS. Prior studies have found that the HINTS examination can outperform early diffusion-weighted MRI for the detection of stroke in patients with AVS, with MRI missing approximately 12% of strokes within the first 48 h of the onset of symptoms (7). Additionally, one study reported that MRI achieved a sensitivity of 83% for diagnosing acute stroke, which was substantially higher than computed tomography (CT), but it still failed to identify a small set of patients with clinically validated cerebrovascular events (16). In contrast, HINTS demonstrated high sensitivity for central pathology (7). However, the successful utilization of the HINTS examination requires the recognition of subtle eye movement abnormalities, such as nystagmus and skew deviation. Studies have emphasized the difficulty of translating HINTS performance into emergency department practice, specifically for clinicians with limited neuro-otologic training and minimal experience with assessing eye movements (17). Educational interventions alone would not be able to fully address the challenges associated with eye movement assessment. Although training programs that include targeted training and vestibular curricula can improve clinician performance in HINTS examinations, the accurate identification of subtle eye movement abnormalities is highly reliant on the expert’s level of experience and consistent exposure to this practice (18). Studies have shown that the HINTS examination requires moderate training to achieve a high diagnostic accuracy, and many clinicians have limited opportunities to develop sufficient proficiency in such skills (19).

Several approaches have been proposed to improve the objective evaluation of eye movements in patients with AVS. Video oculography (VOG) is currently considered a reference standard for the quantitative analysis of eye movements and has demonstrated high performance in detecting eye movement abnormalities that are relevant to the HINTS test. In particular, VOG HINTS (vHINTS) has achieved high diagnostic accuracy for detecting stroke, but it requires specific hardware, specialized expertise, and immense funding (20). Recently, one study investigated the feasibility of performing the HINTS examination using a mixed-reality head-mounted display, which has the ability to measure head and eye movements, such as nystagmus and skew deviation (21). This approach provides an efficient and comprehensive assessment of HINTS with the assistance of machine learning, but it is still reliant on specialized wearable hardware, and the headset is not able to automatically determine whether or not the examiner performs the head impulse test accurately. Recent advances made in computer vision have highlighted the potential of smartphone-based ocular motor assessment. One particular study developed ConVNG, a convolutional neural network for tracking pupil movements that enables offline smartphone video nystagmography. Importantly, ConVNG demonstrated slow-phase velocity measurements with an accuracy similar to that of VOG, and it requires only a smartphone camera and no specialized eye-tracking hardware. The authors of the study described this approach as efficient method of addressing accessibility limitations of conventional VOG and enhancing eye movement assessment methods. These findings support the broader concept that widely available smartphone technology can potentially provide clinically meaningful ocular motor measurements (22).

Recently, a smartphone-based HINTS/HINTS+ application that incorporated video recording capabilities and integrated artificial intelligence was assessed in a group of 10 patients who presented vestibular symptoms (16). The application demonstrated high sensitivity in identifying a single patient with central pathology and facilitated evaluation of nystagmus, skew deviation, and saccades during head-impulse testing. However, as this was a proof-of-concept study, several limitations existed, such as only one patient with a central lesion, and application of the HINTS examination to two patients with BPPV, which HINTS examination does not apply to. Additionally, the application did not include an automated eye-tracking pipeline. Conversely, this EyePhone study focuses specifically on evaluating the feasibility of quantifying induced vertical misalignment using an eye-tracking pipeline.

The EyePhone application utilizes an accessible and widely available smartphone, and it does not require additional hardware. Our current study should not be considered as an individual assessment of skew deviation, but instead, as a component of a broader attempt to develop a smartphone-based application that is capable of evaluating all aspects of the HINTS examination. As briefly mentioned earlier in this section, our previous studies demonstrated the feasibility of smartphone-based head impulse testing (11) and the quantification of nystagmus (13), and this present study supplements these previous efforts by evaluating the application’s feasibility in detecting and quantifying skew deviation. These studies suggest that the EyePhone application can potentially offer an accessible and reproducible approach to the evaluation of eye movement. Importantly, it can pave the way for a reduced dependence on specialized equipment as well as expertise.

The EyePhone application showed a high correlation between its measurements and the design induced skew deviations. Consequently, the application may potentially be valuable for monitoring responses to varying neurological conditions associated with vertical misalignment, such as multiple sclerosis, Myasthenia Gravis, or cranial nerve palsies like trochlear nerve palsy and strabismus (23, 24). Additionally, by observing changes in vertical misalignment across different eye positions, this application may potentially serve as an adjunctive tool following further validation with a patient group presenting with pathologic symptoms of skew deviation, representative of acute vestibular syndrome or stroke.

It should also be noted that the purpose of the EyePhone application is not to replace traditional methods, such as video oculography (VOG), but rather to explore its ability to serve as a method to improve access in emergency settings where expensive technology (ICS video goggles, Natus, WI ~ $40,000) and expertise is limited. Although skew deviation does not necessarily require specialized equipment for detection, the quantification of vertical misalignment is dependent on the examiner. With a smartphone application, there are many advantages in terms of measurement standardization, reproducibility, and long-term monitoring of eye movements. Previous work from our group highlighted the feasibility of using EyePhone to quantify the vestibulo-ocular reflex during head impulse testing (11), and another study demonstrated the feasibility of the application’s ability to detect and quantify nystagmus (13). These studies represent incremental steps towards a unified and accessible method of assessing objective eye movements. This current study focuses primarily on the final component of the HINTS examination (skew deviation) and investigates the feasibility of the application’s ability to quantify vertical ocular misalignment. Accordingly, the potential value and contributions of EyePhone lie not solely in the evaluation of skew deviation, but in its ability to contribute to a comprehensive pipeline for objective evaluation of eye movement findings. With further validation and a larger, clinically relevant sample size, the EyePhone application may potentially improve diagnostic outcomes and accessibility to eye movement assessment.

This feasibility study utilized a sample size of 10 healthy volunteers, which was a convenient sample under controlled experimental conditions. This was not designed to establish clinical diagnostic performance or validation, and the results of this study do not suggest any absolute clinical implications. That being said, this sample size may limit the generalizability of the findings to individuals with different pathologies that could affect vertical misalignment measurements. Additionally, repeated measurements within the same participant group may have inflated correlation and ROC-based performance estimates. Due to the fact that each subject was only tested once, our design does not allow for test–retest variability analysis. Moreover, the inclusion of healthy participants can also introduce a potential bias as patients in emergency departments may show more complex eye movements due to the underlying pathology. As the study was performed under standardized experimental conditions, variability that is commonly encountered in emergency department populations, such as differences in facial anatomy, eyelid morphology, recording conditions, and coexisting neurologic or ocular abnormalities, were not reflected in this study design. Additionally, in this study, we simulated skew deviation using targets placed on the wall, which allowed precise control over the involved variables. However, this controlled setup may not be a full replication of the clinical environment in the emergency departments. We utilized separated targets to simulate the skew deviation, which while effective, does not encapsulate the many variations in skew that are presented in clinical settings.

It is important to note that the current methodology has areas of improvement, specifically for smaller vertical misalignments as the application occasionally misinterprets slight deviations as noise. The Bland–Altman analysis in Figure 5 emphasizes this by indicating the application’s slight tendency to overestimate true deviations, and the limits of agreement show variability in the accuracy of measurement.

## 5 Conclusion

The application demonstrated preliminary feasibility metrics, including an AUC of 0.90 for discriminating skew deviations at clinically significant diopter values. However, these findings are limited to simulated skew deviations in a group of healthy volunteers and should not be considered as evidence of clinical diagnostic performance. Accordingly, the next phase of our work will evaluate the application’s performance in a real-world setting on heterogeneous patient populations with pathologic skew deviations.

## Data Availability

The raw data supporting the conclusions of this article will be made available by the authors, without undue reservation.
